# Assessment on CPR Knowledge and AED Availability in Saudi Malls by Security Personnel: Public Safety Perspective

**DOI:** 10.1155/2020/7453027

**Published:** 2020-04-13

**Authors:** Samer A. Al Haliq, Omar M. Khraisat, Mohamed A. Kandil, Mohammed A. Al Jumaan, Faris M. Alotaibi, Fahad S. Alsaqabi, Hussain M. Alajmi, Hany A. Ellouly, Mahmoud A. Al-Haliq, Abdullah Alkhawaldeh, Mohammed ALBashtawy, Sawsan H. Abuhammad

**Affiliations:** ^1^Department of Emergency Medical Care, Imam Abdulrahman Bin Faisal University, Dammam 31441/1982, Saudi Arabia; ^2^Department of Nursing, Al-Ahliyya Amman University, Amman 19328, Jordan; ^3^Department of Coaching and Sport Management, Hashemite University, Zarqa 13115/591504, Jordan; ^4^Department of Nursing, Jerash University, Jerash 26150/130, Jordan; ^5^Department of Nursing, Al Al-Bayt University, Mafraq 25113/130040, Jordan; ^6^Department of Nursing, Jordan University of Science and Technology, Irbid 22110/3030, Jordan

## Abstract

Security personnel are the first ones who attend the scene in the case of out-of-hospital cardiac arrest (OHCA) at malls. Cardiopulmonary resuscitation (CPR) is not enough for those patients; they need an automated external defibrillator (AED) to bring the heart to function normally. This study aimed to assess the current status of CPR and AED knowledge and availability in Saudi malls by security personnel. Using a descriptive design, a study was conducted at seven malls located in the Eastern Province of Saudi Arabia. Two hundred and fifty participants were surveyed using the American Heart Association (AHA) 2015 guidelines to assess CPR and AED knowledge and availability in Saudi malls. The sample mean age was 32.60 years (SD = 10.02), and 87% of participants were working as security personnel. The majority of the participants had not received training about CPR and AED (75.8% and 95.2%, respectively). Common misconceptions are fallen into all categories of CPR and AED knowledge. Correctly answered statements ranged from 7.2% in the compression rate to 24.2% in hand placement. The study results indicated a poor training knowledge of CPR and AED in public settings. Integrating high-quality CPR and AED knowledge within the school and college curricula is a vital need. However, in order to maximize the survival rate, it is important to set laws and legislation adopted by stakeholders and decision makers to advocate the people who try to help, mandate AED installation in crowded places, and mandate teaching hands-only CPR and AED together as a package.

## 1. Introduction

Cardiovascular disease is a leading cause of worldwide mortality, accounting for approximately 17 million deaths yearly [1]. Approximately 50 to 110 per 100,000 out-of-hospital cardiac arrest (OHCA) annual incidence occurs in the United States and Europe populations [2, 3]. In the United States, only 10.4% of OHCA who had received resuscitation from Emergency Medical Services (EMS) survived to hospital discharge [3]. In Saudi Arabia, the cardiovascular disease mortality rate is 37% according to the World Health Organization (WHO) statistics [4]. A study conducted in Riyadh city in Saudi Arabia reported a high mortality rate of 95.8% among adult OHCA patients; this study reported that poor training of cardiopulmonary pulmonary resuscitation (CPR) and nonutilization of an automated external defibrillator (AED) might be the major factors that stand behind the OHCA high mortality rate [5]. Similarly, the study which was conducted among nonmedical individuals in 2018 at Jeddah city, the second largest city in Saudi Arabia, revealed that the participants perceived a lack of knowledge regarding CPR [6].

Emergency Cardiovascular Care (ECC) 2020 impact goals focus on and set an ambitious target to double the cardiac arrest survival rates and out-of-hospital community response [7, 8]. The community forms the first three links (recognition and activation of the emergency response system, immediate high-quality CPR, rapid defibrillation, basic and advanced EMS, advanced life support, and postarrest care) of the American Heart Association (AHA) to improve the survival rates of adult chain of survival of OHCA [9–11].

The chain of survival can be improved through activating the community by increasing the public awareness regarding the importance of early defibrillation [10, 12–14]. The AHA Highlights 2017 recommendations support the community hands-only CPR training to be performed for OHCA adult victims [10]. CPR alone is not enough, and the AEDs are important to restore the normal sinus rhythm and to bring the heart to function normally [12, 15]. Early CPR and AED utilization by the community may assist in life saving, and it is associated with a two- to three-fold increase in survival when compared to victims who had no CPR and AED before the EMS arrival [9, 13, 16]. Additionally, for most victims, AED is not offered until the EMS crews reach the scene, and for every minute delay in defibrillation, the chances of survival decline by 10%, so familiarity with the public access defibrillation (PAD) may enable rapid defibrillation before EMS arrival [17].

Despite the efforts to focus on the utilization of AED, there are still a lot of AED issues; research suggests that the community faces challenges regarding the utilization of AEDs [18]. Moreover, the community is afraid from AEDs that might be dangerous, complicated technically, and difficult to use because of their limited knowledge and familiarity with them [19].

In Saudi Arabia, 2030 Royal vision was built around three themes: a vibrant society, a thriving economy, and an ambitious nation. A vibrant society is supported by an empowering social and health care system. The security person is the first one who attends the scene in the case of OHCA at malls. Thus, this study aimed to assess the need for AED public access that might empower the health care system and help support community safety regarding ECC in public settings following 2030 vision. Specifically, the study aimed to assess the current status of CPR and AED knowledge and availability in Saudi malls by security personnel.

## 2. Materials and Methods

### 2.1. Study Design, Sample, and Setting

A descriptive design was used to assess the current status of CPR and AED knowledge and availability in Saudi malls by security personnel.

The study was conducted over a period of six months in seven major malls located in Dammam and AL Khobar, Eastern Province of Saudi Arabia. The inclusion criteria for this study were all employees in the selected malls, namely, the security guards and administrators who were able to understand written Arabic.

The estimated sample size was calculated using the Power Primer (Cohen, 1992). The test revealed that using a desired power of 0.80, medium effect size (*r* = 0.25), and 0.05 level of significance, the estimated sample size was 200 mall staff. Oversampling was utilized to gain increased understanding and to overcome participant attrition. Thus, 250 mall employees were selected to participate in the study.

### 2.2. Instruments

The study utilized anonymous self-reported questionnaire. The questionnaire had been designed according to the 2015 AHA guidelines and 2017 AHA Highlights, and it was adapted to be used in this study. This questionnaire was utilized by many studies to assess the public knowledge of adult CPR and effective use of AED [10, 11, 15]. The questionnaire was translated and back translated by bilingual PhD holders. A pilot study was conducted to evaluate the clarity and appropriateness of the questionnaire to the Saudi Arabian culture.

The questionnaire included two parts. Part one is a demographic data sheet that includes questions designed to elicit information about participants' demographic characteristics, such as their age, gender, level of education, job title, whether they have received CPR and AED training or not, additional questions regarding information about the source of CPR and AED training, witnessing cardiac arrest cases while working, and AED device availability in the workplace. The second part includes statements to assess the knowledge of adult CPR and AED utilization. It includes whether or not they know the EMS number in Saudi Arabia, steps of CPR performance (depth, rate, hand placement, and compression-ventilation ratio), and the universal steps of AED operation.

### 2.3. Pilot Study

A pilot study was conducted at one of the selected malls to test the instrument's psychometric properties, the time required to complete the questionnaire, and its clarity. Twenty participants completed the questionnaire within 5–15 minutes. The psychometric evaluation of the English version of the questionnaire was evaluated by content validity. However, the internal consistency of the questionnaire was measured using Cronbach's alpha (*α*) coefficient. Reliability revealed an alpha coefficient of 0.71.

### 2.4. Ethical Considerations

A standard code of ethics for participants and the requirements of the Institutional Review Board (IRB) were followed. The study adheres to the “Guidelines for Ethical Research Practice” and was approved by advisory board from the research committee at the university (IRB: 2019-03-155). The study package included an introductory letter explaining the purpose of the study. The participants were informed that their participation was voluntary, they have the right to withdraw from the study at any time without penalty, and that all the information obtained would be treated confidentially and anonymously. A consent form was attached to the questionnaire. All questionnaires and study materials were kept in a secured cabinet in the principal investigator's office.

### 2.5. Data Collection and Procedures

Approval to conduct the study was obtained from the IRB at the university. Data were collected from March 1 to August 30, 2019. Permission was also obtained from the Malls' administration. The participants were approached in the work setting, and the questionnaire was distributed at the end of the work. The researchers and the administration decided when and how to approach possible participants. Participants were informed about the purposes of the study. They were provided with the questionnaire along with a cover letter.

### 2.6. Data Analysis

The data were coded using the SPSS version 21 (SPSS, Inc., Chicago, IL, USA). Data were screened for missing data and outliers. No missing values and outliers were found. To meet the study aim, descriptive statistics was applied to data.

## 3. Results and Discussion

### 3.1. Demographic Data

A total of 250 questionnaires were distributed; 207 (82.8%) were returned. The features of the participants are displayed in Table 1.

### 3.2. CPR and AED Training Status

A majority of 157 (75.8%) participants reported having received no training about CPR. Of all those 20 (9.7%) who were CPR trained, 10 (4.8%) of them included AED training. However, 197 (95.2%) of the participants reported having received no training about AED. In addition, 207 (100%) of the participants reported that the AED device is not available in all working areas, with 14% of the participants stating that they had previously witnessed sudden cardiac arrest as described in Table 1.

### 3.3. Knowledge about CPR and AED

In Table 2, the top misconceptions (fallacies) about the knowledge of adult CPR and AED among participants are presented. It is clear that participants' most common misconceptions are not limited only to one aspect of CPR performance or AED, but fall into all main categories: the compression-ventilation ratio (90.8%), the adult compression rate (92.8%), the adult compression depth (84.5%), the hand placement in adult CPR (75.8%), and the universal steps of AED operation (92.3%). In addition, more than half of the participants (54.1%) did not know the correct EMS number in Saudi Arabia.

This study aimed to assess the current status of CPR and AED knowledge and availability in Saudi malls by security personnel. The findings of the study showed that the majority of the (86%) participants were male; culturally, gender equality of ease of access to resources and job opportunities was still limited in Saudi Arabia, which is explained by a few participants of female security personal accepted to participate in the study. Also, the findings of the study showed that the majority of the participants have a secondary level of education, and the rate of participants who received no CPR training was displayed to be 75.8%. As well as, the participants who received no AED training were found to be 95.2% as shown in Table 1.

Different countries have conducted several studies in their societies to assess the knowledge and awareness regarding CPR and AED utilization [6, 14, 20–26]. CPR training rates in other countries were as follows: in the western region at Jeddah in Saudi Arabia (28.7%) [6] and a similar rate (29%) in Jordan [21]. Additionally, 21% in Hong Kong [24], 35% in Japan [14], 74% in New Zealand [26], 75% in Poland [27], 79.3% in Washington [25], 68% in Australia [23], in Sweden 45% [22], and 40.3% in Turkey [20]. This can be explained by the fact that it is obligatory to have CPR training by the law of Occupational Health and Safety in these countries [6, 14, 20–25].

In Saudi Arabia, the Saudi Heart Association (SHA) and AHA have taken the responsibility of delivering accredited CPR and AED courses. However, unaccredited CPR and AED courses were delivered through lectures, video presentation, workshops, and internal institute training. The results indicated that CPR and AED training might be primarily delivered through unaccredited CPR and AED courses as evidenced in Table 1, and that 14% of participants stated that they had previously witnessed sudden cardiac arrest. Similarly, 15.8% at Jeddah in Saudi Arabia [6], 18.6% in Turkey [20], 19% in Japan [14], and 23.3% in Jordan [21]. Most of the participants witnessed sudden cardiac arrest without performing CPR; this can be explained by the fact that the participants are hesitant to apply CPR and AED universal steps since they do not have enough knowledge to perform CPR and AED effectively [6, 14, 20, 21]. They are also afraid to make a mistake particularly with no AED device available in their workplace [19]. Many studies explained that poor availability, adequacy, distribution of AED, and training might be major factors that stand behind the OHCA high mortality rate [5, 6, 14, 21].

Recognition and activation of the emergency response system is the first link of adult chain of survival of OHCA; however, it is important to know the local EMS number [9–11]. The results indicated that more than half of the participants (54.1%) did not know the correct EMS number in Saudi Arabia. This might refer to the utilization rate of EMS services in Saudi Arabia was very low in comparison to other countries and states [28].

Quality of CPR concepts were evaluated: compression depth and rates and hand placement. The rates were found to be significantly higher in participants who received CPR training than in those who did not receive CPR training. The results indicated that the participants held a considerable number of misconceptions about CPR and AED utilization, and they had insufficient knowledge about the core and principles of CPR and AED universal operation steps. These results are partially consistent with previous studies with regard to poor knowledge of CPR and AED utilization [5, 6, 20, 21, 24, 26, 29]. However, some aspects were different in Saudi Arabia. This might refer to the status of CPR and AED utilization, and the structured community safety programs for CPR and AED awareness and public access are not available in Saudi Arabia. On top, CPR and AED services are limited to some hospitals as training courses. Further research to examine the availability, adequacy, distribution, and need of CPR and AED training in Saudi Arabia is strongly recommended. Another explanation for the poor knowledge of CPR and AED could be the lack of education content about it in school and college curricula. However, in other countries, it is obligatory to have CPR and AED courses before driving license and they are integrated into secondary schools curricula in some courtiers such as Austria, Japan, Norway, Hong Kong, and Singapore [14, 20, 23–25].

### 3.4. Limitations

Considering the importance of the studied issue, the study involved a small sample of security personnel from seven malls only, and the findings may not be representative of the status of CPR and AED knowledge among security personnel in other settings.

Moreover, the use of a self-report questionnaire could introduce bias, in that participants might not always give full descriptions of their CPR and AED training experience. Also, this study was limited to one area located in the Eastern Province of Saudi Arabia, which limits the external validity of the findings. Future research should include additional studies with a larger sample size recruited from other community settings such as airports, mosques, and football stadiums.

## 4. Conclusions

The results of this study show that participants have insufficient knowledge about adult CPR and AED universal operation steps. This study indicates the importance of generalizing CPR and AED training to the public; by this means, the rate of witnesses who start CPR can be raised. Moreover, laws and legislation should set an effective practical system supporting CPR and AED training in the public settings, mandate AED installation in crowded places, and teach hands-only CPR and AED together as a package to improve the survival rate and enhance public safety.

Thus, integrating high-quality CPR and AED knowledge within the school and universities curricula is a vital need. More descriptive studies are still needed to gain a comprehensive understanding of the outcomes of CPR and AED training in Saudi community settings.

## Figures and Tables

**Table 1:** The demographic features of the participants.

Characteristics	Result
Age	Mean 32.60 years (SD 10.02)

Gender	
Male	178 (86%)
Female	29 (14%)

Level of education	
Less than secondary education	56 (27.1%)
Secondary education	111 (53.6%)
College education	21 (10.1%)
University education	19 (9.2%)

Job title	
Administrator	27 (13%)
Security personnel	180 (87%)

Receive any training covering CPR	
Yes	50 (24.2%)
No	157 (75.8%)

Receive any training covering AED	
Yes	10 (4.8%)
No	197 (95.2%)

Source of CPR and AED training	
Web/computer	4 (1.9%)
Accredited CPR course	20 (9.7%)
Training books/written materials	1 (0.5%)
Video presentation	1 (0.5%)
Didactic lecture	8 (3.9%)
Internal institute training	16 (7.7%)

Witness cardiac arrest cases while working	
Yes	22 (14%)
No	178 (86%)

AED device availability in workplace	
Yes	0 (0%)
No	207 (100%)

SD = standard deviation; CPR = cardiopulmonary resuscitation; AED = automated external defibrillator.

**Table 2:** Descriptive results of participant's answers on the knowledge of adult CPR and AED.

Item	Question	Item participants answers	
		Correct	Wrong
		Frequency (%)	Frequency (%)
1	The EMS number in Saudi Arabia is? (*T*^B^)	95 (45.9)	112 (54.1)
	(A) 996		
	(B) 997		

2	The adult compression-ventilation ratio for 1 or 2 rescuers is? (*T*^B^)	19 (9.2)	188 (90.8)
	(A) 15 : 2		
	(B) 30 : 2		

3	The adult compression rate is? (*T*^B^)	15 (7.2)	192 (92.8)
	(A) 80–100/min		
	(B) 100–120/min		

4	The adult compression depth is? (*T*^A^)	32 (15.5)	175 (84.5)
	(A) At least 2 inches (5 cm)		
	(B) Less than 2 inches (5 cm)		

5	The hand placement in adult CPR is? (*T*^A^)	50 (24.2)	157 (75.8)
	(A) Two hands on the lower half of the breastbone (sternum)		
	(B) Two hands on the middle of the breastbone (sternum)		

6	When the AED device arrives, what should you do next? (*T*^B^)	16 (7.7)	191 (92.3)
	(A) Attach pads, power on, analyze and follow AED prompts		
	(B) Power on, attach pads, analyze and follow AED prompts		

EMS = Emergency Medical Services; *T*^A^ = true choice A; *T*^B^ = true choice B; CPR = cardiopulmonary resuscitation; AED = automated external defibrillator.

## Data Availability

The datasets used and/or analyzed during the current study are available from the corresponding author on reasonable request.
